# The longitudinal analysis for the association between smoking and the risk of depressive symptoms

**DOI:** 10.1186/s12888-024-05828-7

**Published:** 2024-05-15

**Authors:** Sung Keun Park, Chang-Mo Oh, Eugene Kim, Jae-Hong Ryoo, Ju Young Jung

**Affiliations:** 1 Center for Cohort Studies, Total Healthcare Center, Kangbuk Samsung Hospital, Sungkyunkwan University School of Medicine, Seoul, Korea; 2 Departments of Preventive Medicine, School of Medicine, Kyung Hee University, Seoul, Korea; 3 Department of Orthopaedic Surgery, Kangbuk Samsung Hospital, Sungkyunkwan University School of Medicine, Seoul, Korea; 4 Departments of Occupational and Environmental Medicine, School of Medicine, Kyung Hee University, Seoul, Korea; 5 Total Healthcare Center, Kangbuk Samsung Hospital, Sungkyunkwan University, School of Medicine, Seoul, Korea

**Keywords:** Smoking, Depressive symptoms, CES-D, Nicotine, Urine cotinine

## Abstract

**Background:**

Despite high smoking rate in people with depressive symptoms, there is ongoing debate about relationship between smoking and depressive symptoms.

**Methods:**

Study participants were 57,441 Korean men. We collected their baseline data between 2011 and 2012, and conducted follow-up from 2013 to 2019. They were categorized by smoking status (never: < 100 cigarettes smoking in life time, former: currently quitting smoking, and current smoker: currently smoking), smoking amount (pack/day and pack-year) and urine cotinine excretion. The development of depressive symptoms was determined in CES-D score ≥ 16. Cox proportional hazards model was used to analyze the multivariable-adjusted hazard ratio (HR) and 95% confidence intervals (CI) for depressive symptoms in relation to smoking status, smoking amount, and urine cotinine excretion.

**Results:**

During 6.7 years of median follow-up, the risk of depressive symptoms increased in order of never (reference), former (HR = 1.08, 95% CI: 1.01—1.15) and current smoker (HR = 1.24, 95% CI: 1.16—1.32). Among current smoker, the risk of depressive symptoms increased proportionally to daily smoking amount (< 1 pack; HR = 1.21, 95% CI: 1.13—1.29, and ≥ 1 pack; HR = 1.34, 95% CI: 1.23 – 1.45). This pattern of relationship was consistently observed for pack-year in former smoker and current smoker. Additionally, urine cotinine excretion was proportionally associated with the risk of depressive symptoms.

**Conclusion:**

Exposure to smoking was associated with the increased risk of depressive symptoms. Dose dependent relationship was observed between smoking amount and the risk of depressive symptoms.

**Supplementary Information:**

The online version contains supplementary material available at 10.1186/s12888-024-05828-7.

## Background

There has been growing awareness for the association between smoking and mental illness. People with mental illness are two to three times more likely to smoke, compared with people without mental illness [1, 2]. Additionally, evidence has indicated that smoking rate increases with the severity of mental illness [3]. Although smoking rate has declined over decades due to anti-smoking campaign and tobacco control policies [4], people with mental illness are still difficult to quit smoking due to greater nicotine dependence and withdrawal symptoms [5, 6].

Depression is most common mental illness with a lifetime prevalence ranging from approximately 11–15% [7]. The high co-occurrence of smoking and mental illness arouses the interest on the association between smoking and depression accounting for the major proportion of mental illness. Published literatures have shown that smoking is potentially associated with the increased risk of depression [8–10]. A systematic review for 148 studies indicated that exposure to smoking at baseline led to the increased incidence of later depression [8]. Wu et al. demonstrated that smoking amount was positively associated with the risk of depression in a cross-sectional study [9]. In a systematic review, long-term smoking cessation over 10 years was negatively associated with the risk of depression [10]. These results suggest that smoking is a potential risk factor for depression. However, current evidence supports the bidirectional relationship between smoking and depression. High prevalence of smoking among people with depression suggests that presence of depression has a role in the initiation of smoking [11, 12]. In addition, studies have indicated that people with current or past depression are about twice more likely to smoke than people without depression [5, 13]. Reports for the effect of depression on smoking are based on hypothesis that smoking alleviates depressive symptoms [14, 15]. These results indicate that bidirectional association exists between smoking and depression, rather than unilateral direction. Moreover, some studies showed insignificant association between smoking and the risk of depression, in which confounding factors and causal models acted as risk determinants [16, 17]. Considering the positive, bidirectional, and non-significant relationship between smoking and depression, longitudinal analysis for the effect of smoking on the risk of depression may contribute to establishing the association between smoking and depression.

The aim of the present study is to examine the effect of smoking on the development of depression. We longitudinally assessed the risk of depression according to smoking status, smoking, and urine cotinine excretion amount among 57,441 working aged Korean men.

## Method

### Study participants and exclusion criteria

Relevant clinical and sociodemographic data were obtained from Kangbuk Samsung Health Study (KSHS). KSHS is a cohort study to investigate the medical data of Koreans who have received medical health check-up in Kangbuk Samsung Hospital. Korea’s Industrial Safety and Health law orders that all of Korean employees should receive medical health check-up annually or biennially.

**Fig. 1:**
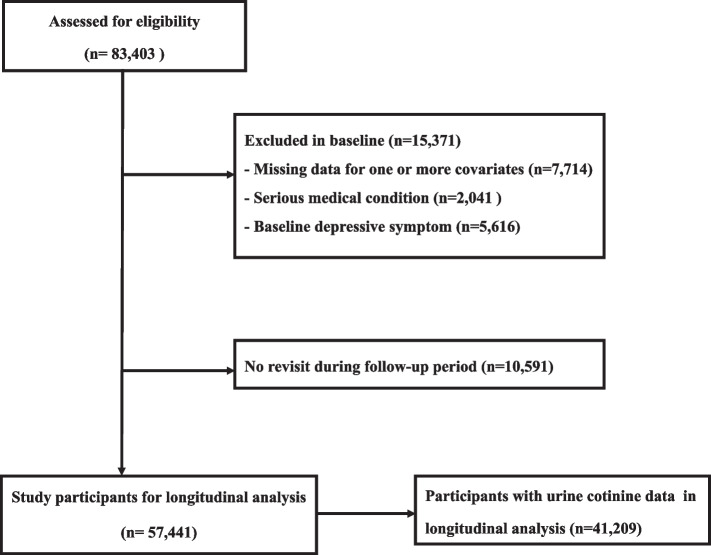
Flow-chart of enrolled study participants

Owing to low smoking rate in women, we included only men in study participants. We initially enrolled 83,403 men who had responded to both smoking related questionnaires and Center for Epidemiological Studies-Depression Scale (CES-D) between March 2011 and December 2012. Among initial 83,403 men, we excluded 7,714 men with missing value in covariate variables (e.g. education level and marital status), and 2,041 men with a history of serious medical diseases (e.g. corone heart disease, stroke, and cancer) that affect smoking and mood. In addition, we further excluded 5,616 men with baseline depressive symptoms (CES-D ≥ 16) and 10,591 subjects who lost to follow-up due to not revisiting. Finally, eligible study participants were 57,441 men who revisited and responded to the CES-D questionnaire from January 2013 to December 2019 (Fig. 1).

### Clinical, smoking and sociodemographic data

Study data consists of medical history assessed by self-administered questionnaire, anthropometric measurements and laboratory measurements. All study participants were asked to respond to a health-related behavior questionnaire, which included the topics of alcohol consumption, smoking and exercise. Smoking pattern was categorized into three status; never, former, and current smoker. Never smoker was defined as those who have smoked less than 100 cigarettes or have never smoked in their lifetime [18]. The smoker was defined as those who have smoked ≥ 100 cigarettes in their lifetime. Out of smokers, subjects who were currently smoking were defined as current smoker, and subjects who were currently quitting smoking were defined as former smoker. The degree of physical activity was evaluated by the Korean-validated version of the International Physical Activity Questionnaire (IPAQ) short form and classified into three categories (low, moderate, and high) according to the guidelines prescribed by the IPAQ core group (http://www.ipaq.ki.se). Questions about marriage, and education were also included in the questionnaire. Because most study participants were married, they were categorized into married and non-married status. In addition, since many of the participants were highly educated with a college or graduate school degree, we used college graduation or higher as a classification criterion for high education. Hypertension was defined as a prior diagnosis of hypertension or having a measured BP ≥ 140/90 mmHg at initial and follow up examinations. Trained nurses measured blood pressure (BP) on sitting position by automatic device (53,000-E2, Welch Allyn, USA) three times after a 5 min rest with at least 30 s interval. Final BP levels were obtained as average of second and third BP measurements [19]. The body mass index (BMI) was calculated by dividing weight (kilograms) by square of height (meters^2^). Diabetes Mellitus (DM) was defined as one of following conditions; fasting glucose ≥ 126 mg/dL, hemoglobin A1 c (HbA1c) ≥ 6.5%, and a prior diagnosis of DM [20].

Blood samples were collected after more than 12 h of fasting and were drawn from an antecubital vein. The fasting serum glucose was measured using the hexokinase method, and HbA1c was measured using an immunoturbidimetric assay with a Cobra Integra 800 automatic analyzer (Roche Diagnostics, Basel, Switzerland). Serum uric acid levels were measured enzymatically using an automatic analyzer Advia 1650 Autoanalyzer, Bayer Diagnostics; Leverkusen, Germany). Urine cotinine excretion was assessed by using the DRI Cotinine Assay (Microgenics Corp., Fremont, CA, USA) and a modular P800 chemistry analyzer (Roche Diagnostics, Tokyo, Japan). The cut-off of urine cotinine excretion was set at 50 ng/mL on the basis of recommendations from Society for Research on Nicotine and Tobacco [21]. Subjects with urine cotinine excretion above 50 ng/ml were divided into three groups by tertile.

### Assessment of depressive symptoms

Depressive symptoms were assessed using the Korean versions of CES-D scale [22]. The CES-D scale is a self-report questionnaire designed to assess the current presence of depressive symptoms in the general population [23]. We used the 4-factors 20-items CES-D scale with scores ranging from 0 to 3, with 0 indicating that the depressive symptoms were experienced rarely and 3 indicating that depressive symptoms were experienced most of the time in the past week. (e.g. “I thought my life had been a failure.” 0 = seldom (not at all or less than 1 day), 1 = sometimes (1 ~ 2 days), 2 = often (3 ~ 4 days), 3 = almost always (5 ~ 7 days)). The presence of depressive symptoms was defied using a classic cutoff of CES-D score (16 or greater) [23].

### Statistical analyses

The baseline parameters among smoking status group (never, former, current) are presented as means ± standard deviation for continuous variables and as proportions for categorical variables. Main clinical characteristics and parameters among study groups were compared using ANOVA for continuous variables and chi-square test for categorical variables.

A Cox proportional hazards model was used to calculate the unadjusted and multivariable-adjusted hazard ratio (HR) and 95% confidence intervals (CI) for depressive symptoms (multivariable adjusted HR [95% CI]) in each smoking group. The models were adjusted for multiple covariates including age, BMI, alcohol intake, hypertension, DM, physical activity, marital status, education. The covariates of the multivariable model were selected from clinical and demographic variables to affect the development of depression. These variables include age, hypertension, DM, BMI, physical activity, alcohol intake, education, marital status, and use of sedative or anxiolytics. The incidence cases, incidence density (incidence of depressive symptom/1,000 person-years), person years of each group were calculated. The proportional hazards assumption was confirmed by log–log plots and Schoenfeld residual test. To verify multicollinearity between variables, we analyzed Variance Inflation Factor (VIF), and it was confirmed that there were no variables with VIF greater than 10.

We additionally analyzed the risk of depressive symptoms in relation to daily smoking amount, pack-year, and urine cotinine excretion. In current smoker, the risk of depressive symptoms was evaluated according to daily smoking amount (never, < 1 pack/day, and ≥ 1 pack/day) and pack-year (never, < 10 pack-year, 10 ≤  ~  < 20 pack-year, and ≥ 20 pack-year). In former smoker, the risk of depressive symptoms was evaluated according to pack-year (never, < 10 pack-year, 10 ≤  ~  < 20 pack-year, and ≥ 20 pack-year).

Urine cotinine excretion was categorized into four groups (< 50 ng/ml, tertile 1: 50–618 ng/ml, tertile 2: 619–1316 ng/ml, and tertile 3: 1317–9739 ng/ml). Because urine cotinine excretion was measured in only 79.3% of study participants, analysis for urine cotinine excretion was conducted for 41,209 men (Fig. 1). Trend analysis was conducted for median value of current smoking amount, pack-year and urine cotinine excretion.

All statistical analyses were performed using R 4.1.3 (R Foundation for Statistical Computing, Vienna, Austria), and a value of *P* < 0.05 (two-sided) was considered statistically significant in all analyses.

## Result

The baseline clinical and sociodemographic characteristics among smoking status groups are presented in Table 1. Our study participants were dominated by working aged Korean men with an average age of 39.6 ± 6.8 years old. While the proportion of never smoker was 16.7%, the proportion of former smokers and never smoker was 43.1% and 40.2%, respectively. Compared to never smokers, current and former smokers had the higher levels in age, alcohol intake, BMI, and the prevalence of hypertension and DM. The baseline CES-D score was highest in current smoker, followed by former and never smoker. During 6.7 years of median follow-up period, depressive symptoms (CES-D ≥ 16) developed in 9,031 participants, presenting the highest incidence in current smoker (17.3%). Supplementary Table 1 shows the comparison of clinical characteristics between participants with follow-up (*n* = 57,441) and participants lost to follow-up (*n* = 10,591). Compared with participants with follow-up, participants lost to follow-up were characterized by high levels of age, alcohol intake, proportion of DM and hypertension, smoking pack-year, and CES-D score.

**Table 1:** Clinical characteristics of study participants according to the smoking status

Characteristics	Never	Former	Current	*P*-value
number	9,591	24,737	23,113	
Age (year)	37.8 ± 7.0	40.3 ± 7.1	39.7 ± 6.2	< 0.001
High education (%)	85.5%	81.4%	75.8%	< 0.001
Married (%)	79.1%	88.9%	85.0%	< 0.001
Average alcohol use (g/day)	13.7 ± 18.0	18.5 ± 23.2	25.7 ± 27.6	< 0.001
High physical activity (%)	16.4%	19.3%	15.7%	< 0.001
Diabetes Mellitus (%)	3.2%	4.6%	5.4%	< 0.001
Hypertension (%)	12.4%	15.1%	14.0%	< 0.001
BMI (kg/m^2^)	24.1 ± 2.9	24.4 ± 2.8	24.6 ± 3.0	< 0.001
Use of Sedative or anxiolytics (%)	0.1%	0.2%	0.2%	0.056
Smoking pack-year	0	6.6 ± 8.3	12.8 ± 8.4	< 0.001
Urinary cotinine ≥ 50 ng/ml (%)	0.1%	4.7%	87.7%	< 0.001
Baseline CESD score	4.5 ± 4.1	4.6 ± 4.0	5.1 ± 4.1	< 0.001

Continuous variables are expressed as mean (± SD), and categorical variables are expressed as number (percentage (%))

*BMI* body mass index, *CESD* Center for Epidemiologic Studies Depression

Table 2 shows the unadjusted and the multivariable adjusted HR and 95% CI for depressive symptoms according to the groups of smoking status. In multivariable adjusted analysis, the risk of depressive symptoms increased in order of current smoker (HR = 1.24, 95% CI: 1.16—1.32), former smoker (HR = 1.08, 95% CI: 1.01—1.15), compared with never smoker. This result suggests that current smoker and former smoker are more significantly associated with the increased risk of depressive symptoms than never smoker.

**Table 2:** Hazard Ratio (HR) and 95% confidence intervals (CI) for depressive symptom according to smoking status

- Smoking status	Never	Former	Current
Number	9,591	24,737	23,113
Unadjusted HR	1.00 (Reference)	1.04 (0.97 – 1.11)	1.22 (1.15 – 1.30)
Multivariable- Adjusted HR	1.00 (Reference)	1.08 (1.01—1.15)	1.24 (1.16 – 1.32)
Incidence density	23.4	25.3	29.6
Person year	54,195	149,254	135,013
case [n, (%)]	1,266 (13.2%)	3,772 (15.2%)	3,993 (17.3%)

Adjusting covariates: age, hypertension, diabetes mellitus, physical activity, alcohol intake, education, marital status, use of sedative or anxiolytics and BMI

Incidence density: incidence of depressive symptom per 1,000 person-years

In Table 3, the risk of depressive symptoms was assessed in relation to the levels of smoking amount categorized by daily smoking (pack per day) in current smoker, and pack-year in current smoker and former smoker. The risk of depressive symptoms increased with daily smoking amount (never; reference, < 1 pack; HR = 1.21, 95% CI: 1.13—1.29, and ≥ 1 pack; HR = 1.34, 95% CI: 1.23 – 1.45). This pattern of relationship was identically observed in pack-year of current smoker (never; reference, < 10 pack-year; HR = 1.18, 95% CI: 1.10—1.27, 10 ≤  ~  < 20 pack-year; HR = 1.27, 95% CI: 1.18 – 1.37, and 20 ≤ pack-year; HR = 1.38, 95% CI: 1.25 – 1.52) and former smoker (never; reference, < 10 pack-year; HR = 1.06, 95% CI: 1.00—1.14, 10 ≤  ~  < 20 pack-year; HR = 1.13, 95% CI: 1.03 – 1.25, and 20 ≤ pack-year; HR = 1.20, 95% CI: 1.04 – 1.38). These results indicate that proportional relationship exists between smoking amount and the risk of depressive symptoms.

**Table 3:** Hazard Ratio (HR) and 95% confidence intervals (CI) for depressive symptom according to the levels of smoking amount

Smoking status	Levels of smoking amount				P for trend
**- Current smoker (pack/day)**	Never	< 1	≥ 1		
Number	9,591	16,879	6,324		
Unadjusted HR	1.00 (Reference)	1.21 (1.13 – 1.29)	1.29 (1.19 – 1.40)		< 0.001
Multivariable- Adjusted HR	1.00 (Reference)	1.21 (1.13—1.29)	1.34 (1.23 – 1.45)		< 0.001
Incidence density	23.4	29.1	31.0		
Person year	54,195	100,092	34,921		
case [n, (%)]	1,266 (13.2%)	2,912 (17.3%)	1,081 (17.3%)		
**- Current smoker (pack-year)**	Never	< 10	10 ≤ ~ < 20	20 ≤	
Number	9,591	9,865	8,520	4,728	
Unadjusted HR	1.00 (Reference)	1.22 (1.14 – 1.31)	1.23 (1.14 – 1.32)	1.24 (1.14 – 1.36)	< 0.001
Multivariable- Adjusted HR	1.00 (Reference)	1.18 (1.10—1.27)	1.27 (1.18 – 1.37)	1.38 (1.25 – 1.52)	< 0.001
Incidence density	23.4	29.3	29.8	29.9	
Person year	54,195	59,259	50,006	25,748	
case [n, (%)]	1,266 (13.2%)	1,735 (17.6%)	1,489 (17.5%)	769 (16.3%)	
**Former smoker (pack-year)**	Never	< 10	10 ≤ ~ < 20	20 ≤	
Number	9,591	17,741	4,846	2,150	
Unadjusted HR	1.00 (Reference)	1.04 (0.97 – 1.11)	1.02 (0.93 – 1.12)	0.99 (0.87 – 1.13)	0.861
Multivariable- Adjusted HR	1.00 (Reference)	1.06 (1.00—1.14)	1.13 (1.03 – 1.25)	1.20 (1.04 – 1.38)	0.002
Incidence density	23.4	25.5	25.1	23.6	
Person year	54,195	109,492	28,334	11,427	
case [n, (%)]	1,266 (13.2%)	2,792 (15.7%)	710 (14.7%)	270 (12.6%)	

Adjusting covariates: age, hypertension, diabetes mellitus, physical activity, alcohol intake, education, marital status, use of sedative or anxiolytics and BMI (1 pack = 20 cigarettes)

Incidence density: incidence of depressive symptom per 1,000 person-years

In Table 4, the risk of depressive symptoms was evaluated according to the tertile levels of urine cotinine excretion. Compared with reference group (urine cotinine < 50 ng/ml), all of tertile groups with urine cotinine ≥ 50 ng/ml had the slight increase in the risk of depressive symptoms (never; reference, tertile 1; HR = 1.09, 95% CI: 1.02 – 1.17, tertile 2; HR = 1.17, 95% CI: 1.09 – 1.25, and tertile 3; HR = 1.08, 95% CI: 1.00 – 1.15). Considering that exposure to smoking is reflected by urine cotinine excretion, exposure to smoking may be associated with the increased risk of depressive symptoms.

**Table 4:** Hazard Ratio (HR) and 95% confidence intervals (CI) for depressive symptom according to tertile levels of urinary cotinine

- Urinary Cotinine	< 50	Tertile 1	Tertile 2	Tertile 3	P for trend
Number	26,702	5,730	5,729	5,724	
Range of urinary cotinine (ng/ml)	< 50	50–618	619–1316	1317–9739	
Unadjusted HR	1.00 (Reference)	1.10 (1.03 – 1.18)	1.18 (1.11 – 1.26)	1.10 (1.03 – 1.18)	< 0.001
Multivariable- Adjusted HR	1.00 (Reference)	1.09 (1.02—1.17)	1.17 (1.09 – 1.25)	1.08 (1.00 – 1.15)	0.002
Incidence density	26.2	29.4	31.5	29.5	
Person year	162,612	34,948	34,457	34,250	
case [n, (%)]	4,256 (15.9%)	1,026 (17.9%)	1,084 (18.9%)	1,009 (17.6%)	

Adjusting covariates: age, hypertension, diabetes mellitus, physical activity, alcohol intake, education, marital status, use of sedative or anxiolytics and BMI

Incidence density: incidence of depressive symptom per 1,000 person-years

## Discussion

In the present study, former smoking and current smoking were associated with the increased risk of depressive symptoms among working aged Korean men, compared with never smoking. Even in given smoking status, the risk of depressive symptoms increased with the levels of smoking amount. These results suggest that exposure to smoking is a risk factor for depressive symptoms. In addition, it is postulated that smoking amount is proportionally associated with the risk of depressive symptoms.

Our results are supported by those of previous studies showing the adverse effect of smoking on the development of depressive symptoms. In a systematic review for 148 prospective studies, over a third of the studies demonstrated that smoking exposure at baseline was associated with later depression, suggesting the significant association between prolonged smoking and increased susceptibility to depression [8]. Depression and drug dependence including smoking may be associated with alteration in some of same neurotransmitter, and in particular, with alteration of neurotransmitter function in limbic-related brain structures [14]. Prolonged smoking can lead to smoking dependence that is linked to depression by some shared neurobiology [14]. These reports underline the impact of smoking duration on the development of depressive symptoms that lacks in our study. Therefore, it should be noted that the development of depressive symptoms is affected by not only smoking amount but also smoking duration. In addition, evidence from previous studies has suggested that smoking per se is a risk factor for depressive symptoms regardless of smoking amount. In a Mendelian randomization study for 462,690 participants from the UK Biobank, smoking was a significant risk factor for depression (OR: 1.99 [1.71–2.32) across both lifetime smoking and smoking initiation [24]. A recent cross-sectional study demonstrated that depression was more significantly associated with previous smokers (OR: 1.25 [1.05–1.48]) and occasional smokers (OR: 1.84 [1.39–2.45]), compared with never smokers [9]. In addition, longitudinal analyses showed that daily smoking increased the risk for subsequent development of depression among adolescents [25, 26]. These results solidify the clinical significance of smoking behavior as a potential risk factor for depression. Adverse effect of smoking on depressive symptoms emphasizes the importance of cessation of smoking in maintaining mental health. Recent evidence suggests that smoking cessation was associated with decreased risk of depression, and longer duration of smoking cessation led to the lower risk of depression [9]. Quitting smoking may be imperative in maintaining mental health as well as physical health.

The responses of nervous system and neurotransmitters to smoking may be explanations for the association between smoking and development of depressive symptoms. It has been proposed that smoking makes people vulnerable to depressive symptoms through adversely affecting neurocircuitry. Nicotine exposure gives rise to the dysregulation in hypothalamic–pituite–adrenal system that leads to hypersecretion of cortisol and disturbance of monoamine oxidase [14], involved in regulating stress response. Cessation of smoking resulted in the normalization of these adverse responses by nicotine exposure [27]. In addition, evidence indicates that chronic smoking causes alteration in the activity of neurotransmitter systems associated with regulating the biological and psychological reactions to stressors [28]. Prolonged smoking may continue to sensitize neurobiological stress response systems, attenuating adaptive ability to outside stress [29]. These detrimental alterations in nervous systems may cause mental and psychological damage, which predisposes smokers to depression.

Self-reported questionnaire has been commonly used to evaluate smoking status and smoking amount in epidemiological studies. However, it is recognized that reliance on self-reported questionnaire can generate the possibility of recall bias. Thus, we used urine cotinine excretion as a dependent variable to obtain the more objective data for smoking. In the analysis, the risk of depressive symptoms slight increased among participants with urine cotinine excretion ≥ 50 ng/ml (≥ teritle 1). This result reinforces the causative relationship of smoking with depressive symptoms. Interestingly, the magnitudes of HR for depressive symptoms in relation to urine cotinine levels are relatively modest, compared with those in relation to smoking status and smoking amount assessed by self-reported questionnaire. A plausible explanation for this finding is a feature of urine cotinine. Although urine cotinine excretion is useful in assessing the validity of self-reported questionnaire, there is a possibility of misclassification error. Urine cotinine excretion can misclassify never smoker with passive smoking as current smoker and former smoker as never smoker [30]. As aforementioned, the effect of smoking on depression is closely linked to the smoking duration, in which prolonged stimulation of smoking on nervous systems acts in development of depression [28, 29]. Therefore, urine nicotine concentration at a specific time doesn’t seem to entirely reflect the effect of prolonged smoking on depression.

Our study hypothesis was not entirely reliant on unilateral relationship of smoking with the development of depressive symptoms. We concede self-medication hypothesis that the presence of depressive symptoms leads to smoking to alleviate depressive symptoms [15, 16]. Although smoking is a potential risk factor for the development of depressive symptoms, depressive symptoms may be a factor associated with increase in smoking rate and smoking amount. Previous studies have also demonstrated the bilateral relationship between smoking and depressive symptoms [8–12]. Smoking may temporarily mitigate stress and depressive symptoms through stimulating nervous system [14, 15]. However, prolonged smoking results in smoking dependence, which gives rise to mental and psychological damage, increasing the susceptibility to depressive symptoms [31].

In the present study, several limitations should be recognized.

First, evaluation for depressive symptoms was performed through only CES-D scale. Although CES-D scale has been widely used in epidemiological studies, CES-D scale is not a gold standard in diagnosing depressive symptoms. Therefore, we acknowledge the possibility for the underestimation or overestimation of depressive symptoms. Second, self-reported questionnaire was used in assessing smoking status and smoking amount. Self-reported questionnaire can be affected by recall bias, which leads to difference from real smoking status and smoking amount. Third, it is impossible to entirely consider potential confounders like sleep patterns, childhood experiences, and social support to affect the development of depressive symptoms. That’s because of limitations in our raw data including high non-response rate for sleep pattern (> 50%), and absence of questions for childhood experiences and social support in questionnaires.

## Conclusion

In summary, the study showed that exposure to smoking was significantly associated with the increased risk of depressive symptoms among working aged Korean men. In particular, heavier smoking amount resulted in the higher risk for depressive symptoms. These results suggest that smoking is a significant risk factor for the development of depressive symptoms. As an effort to ameliorate the burden of depression, it is warranted to recommend cessation of smoking and at least reducing smoking amount.

### Supplementary Information

Supplementary Material 1.

## Data Availability

The data that support the findings of this study are available from Kangbuk Samsung Cohort Study, but restrictions apply to the availability of these data, which were used under license for the current study, and so are not publicly available. Data are however available from the authors upon reasonable request and with permission of Kangbuk Samsung Cohort Study.
