# Supplementary material for: The longitudinal analysis for the association between smoking and the risk of depressive symptoms
Source: BMC Psychiatry. 2024 May 15;24:364. doi: 10.1186/s12888-024-05828-7 (PMC11094926; doi:10.1186/s12888-024-05828-7)
Supplement: Supplementary file 1 — Supplementary Material 1. [file 12888_2024_5828_MOESM1_ESM.docx]

**Supplementary Table 1.** Clinical characteristics of study participants according to the follow-up loss

| **Characteristics** | **Follow-up** | **Follow-up loss** | **P value** |
| --- | --- | --- | --- |
| number | 57,441 | 10,591 |  |
| Age (year) | 39.6 ± 6.8 | 44.5 ± 10.0 | < 0.001 |
| High education (%) | 79.8% | 73.9% | <0.001 |
| Married (%) | 85.7% | 86.2% | 0.149 |
| Average alcohol use (g/day) | 20.6 ± 24.8 | 25.6 ± 31.8 | <0.001 |
| High physical activity (%) | 17.4% | 22.1% | <0.001 |
| Diabetes Mellitus (%) | 4.7% | 8.9% | <0.001 |
| Hypertension (%) | 14.2% | 19.8% | <0.001 |
| BMI (kg/m^2^) | 24.5 ± 2.9 | 24.5 ± 2.9 | 0.458 |
| Use of Sedative or anxiolytics (%) | 0.2% | 0.4% | 0.003 |
| Smoking (%) |  |  |  |
| Never | 9,591 (16.7%) | 2,366 (22.3%) |  |
| Former | 24,737 (43.1%) | 3,673 (34.7%) |  |
| Current | 23,113 (40.2%) | 4,552 (43.0%) |  |
| Smoking pack year | 8.0 ± 8.9 | 11.9 ± 12.4 | < 0.001 |
| Urinary cotinine ≥ 50 ng/ml (%) | 37.5% | 40.7% | <0.001 |
| Baseline CES-D score | 4.8 ± 4.1 | 5.3 ± 4.2 | <0.001 |

Continuous variables are expressed as mean (±SD), and categorical variables are expressed as number (percentage (%)).

BMI: body mass index, CESD: Center for Epidemiologic Studies Depression
